# Simultaneous LC–MS Profiling of Bioactive Ecdysteroids in Nutrient-Dense Plant Sources and Dietary Supplements

**DOI:** 10.3390/molecules31071090

**Published:** 2026-03-26

**Authors:** Velislava Todorova, Stanislava Ivanova, Raina Ardasheva, Kalin Ivanov

**Affiliations:** 1 Department of Pharmacognosy and Pharmaceutical Chemistry, Faculty of Pharmacy, Medical University of Plovdiv, 4002 Plovdiv, Bulgaria; 2 Research Institute, Medical University of Plovdiv, 4002 Plovdiv, Bulgaria; 3 PERIMED-2, BG16RFPR002-1.014-0007, Central District, Vasil Aprilov Blvd. 15A, 4002 Plovdiv, Bulgaria; 4 Department of Medical Physics and Biophysics, Faculty of Pharmacy, Medical University of Plovdiv, 4002 Plovdiv, Bulgaria

**Keywords:** phytoecdysteroids, ecdysterone, turkesterone, LC–MS, superfoods, dietary supplements, sport, validation, quality control

## Abstract

Phytoecdysteroids have garnered increasing interest due to their broad biological and pharmacological properties. The present study reports on the development and validation of a reliable liquid chromatography–mass spectrometry method for the detection and quantification of 20-hydroxyecdysone, turkesterone, and ponasterone. The optimized procedure improved ionization efficiency and chromatographic resolution through gradient elution using 0.1% formic acid in water and acetonitrile. Data acquisition in selective ion monitoring modes ensured high analytical precision, reproducibility, and sensitivity. The method demonstrated excellent linearity, accuracy, repeatability, and low detection limits, making it suitable for routine phytochemical and quality control applications. Application of the method to extracts from nutrient-rich superfoods, including kaniwa, spinach, quinoa, and asparagus, confirmed these plants as natural sources of phytoecdysteroids. Additionally, thirteen commercially available dietary supplements labeled as containing extracts of *Rhaponticum carthamoides*, *Cyanotis arachnoidea*, *Ajuga turkestanica*, or ecdysteroids were analyzed. Several products standardized to 80–95% ecdysterone contained substantially lower amounts than declared, with measured 20-hydroxyecdysone levels ranging from below the limit of detection to approximately 50 mg per capsule, whereas some non-standardized products exhibited moderate to high levels, reaching up to approximately 105 mg per capsule. Variability in turkesterone content was also observed among products marketed as standardized extracts. The method provides a simple, reliable, and accessible approach for the quantitative analysis of major phytoecdysteroids in complex plant matrices and dietary supplements. Its implementation may support phytochemical research, routine quality control, and anti-doping monitoring of ecdysteroid-containing products.

## 1. Introduction

Phytoecdysteroids (PEs) are naturally occurring bioactive compounds synthesized by plants to protect themselves against herbivorous insects. They belong to the ecdysteroid family of polyhydroxylated steroids. Structurally, ecdysteroids posses a cyclopentanoperhydrophenanthrene ring with a β-oriented side chain at C-17 [1,2]. Their multiple hydroxyl groups and variable substituents result in considerable structural diversity. PEs have been identified in a wide range of organisms, including algae, fungi, ferns, gymnosperms, and angiosperms, with over 500 distinct compounds isolated from more than 100 terrestrial plant species [1]. Some ecdysteroids such as ecdysone, 20-hydroxyecdysone (20-HE), makisterone A, and ajugasterone C occur in both the plant and animal kingdoms [2,3]. In insects, ecdysteroids function as steroid hormones regulating molting and development, whereas in plants, they are considered secondary metabolites, likely involved in defense mechanisms against herbivores [4]. PEs have been isolated from various plant sources, including the leaves of *Podocarpus nakaii* Hayata (Podocarpaceae), the bark of *Podocarpus elatus* R.Br. ex Endl., the roots of *Achyranthes fauriei* H.Lév. & Vaniot (Amaranthaceae), etc. [5]. The most common PEs found in plants include 20-HE, ajugasterone C, turkesterone (TS), polypodin B, and ponasterones A-C [6,7]. These PEs also present in edible plants, such as spinach, sugar beet, and quinoa seeds, making them of nutritional and pharmacological interest [6]. The concentration of PEs varies depending on plant species, organ, and environmental conditions [1]. PEs may exist in free or conjugated forms—often bound to organic acids, sugars, or sulfates—resulting in substantial chemical diversity. In recent years, numerous studies have revealed significant advances in understanding the biological activity, structural diversity, and pharmacological potential of plant-derived bioactive compounds, with particular emphasis on PEs [8,9]. Furthermore, plants rich in PEs have been used as adaptogens, antioxidants, and tonics and for enhancing muscle development and physical performance [10,11,12].

Ecdysterone has been monitored by the World Anti-Doping Agency (WADA) since 2020 because of its suspected anabolic properties and potential to enhance athletic performance in both professional and non-professional contexts [13,14]. In this context, accurate quantification of PEs is essential for doping control, as it enables reliable assessment of athlete exposure, differentiation between dietary intake and supplementation, and consistent interpretation of analytical results in anti-doping testing. Commonly described as a “natural anabolic agent,” 20-HE has attracted considerable attention in sports nutrition research [15]. Nowadays, its use has expanded globally due to reported adaptogenic and ergogenic properties [12,15,16,17,18]. Extracts of *R. carthamoides* and related ecdysteroid-containing plants, as well as their secondary metabolites, such as 20-HE and TS, have been associated with improvements in athletic performance [19,20,21,22]. The global dietary supplement (DS) market has expanded rapidly in recent years, reaching an estimated USD 209.52 billion in 2025, and it is projected to grow to USD 393.56 billion by 2033, driven by increasing health awareness, changing consumer lifestyles, and a greater focus on preventive healthcare [23]. Among DSs marketed to enhance physical strength, ecdysteroids are included for their anabolic and performance-supporting effects [24,25]. The principal botanical sources of 20-HE and TS used in DSs include *C. arachnoides*, *A. turkestanica*, and *R. carthamoides* [18,24]. However, the regulatory status of 20-HE remains controversial because it also occurs naturally in commonly consumed foods, such as spinach and quinoa [18,26,27,28]. The increasing consumption of plant-based DSs further highlights the need for reliable analytical control [25,29,30,31]. The inclusion of foods such as spinach and quinoa may, therefore, contribute not only to nutritional adequacy but also to the intake of naturally occurring bioactive compounds, including ecdysteroids [32].

The structural variability of these compounds has led to the use of different analytical approaches for their separation and quantification. Among the most commonly applied techniques are thin-layer chromatography, gas chromatography, high-performance liquid chromatography (HPLC), and supercritical fluid chromatography [33,34]. Nuclear magnetic resonance remains the primary tool for structural elucidation, while HPLC coupled with mass spectrometry (HPLC-MS) provides high sensitivity and selectivity for ecdysteroid detection. In contrast to gas chromatography–mass spectrometry (GC–MS), liquid chromatography–mass spectrometry (LC–MS) does not require derivatization, thereby simplifying sample preparation and reducing potential artifacts. However, many analytical approaches present certain limitations, including the need for derivatization, relatively long analysis times, and limited capability for simultaneous detection of multiple PEs. These constraints may reduce their suitability for routine analysis and high-throughput quality control applications. Simultaneous quantification of the major phytoecdysteroids 20-HE, TS, and ponasterone A (PA) in diverse plant matrices and commercial dietary supplements remains limited, particularly using rapid and cost-effective analytical platforms suitable for routine quality control. Therefore, the present study aims to develop and validate a rapid and reliable LC-MS method for the simultaneous quantification of three phytoecdysteriods (20-HE, TS, and PA) in plant extracts and commercial DSs.

## 2. Results and Discussion

**Figure 1:**
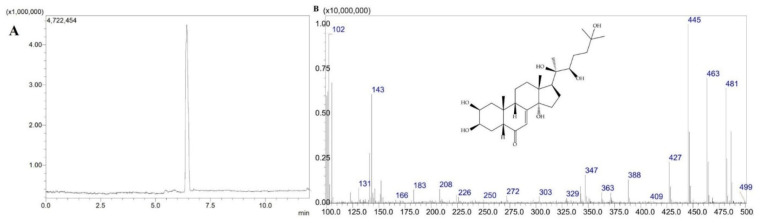
TIC chromatogram (**A**) and mass spectrum of 20-HE (**B**).

**Figure 2:**
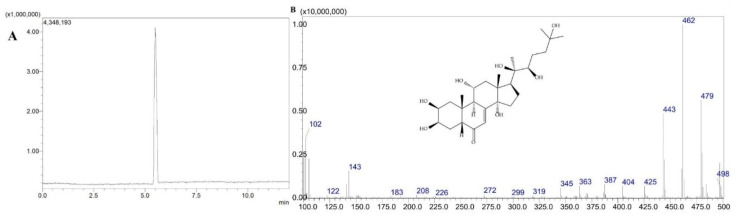
TIC chromatogram (**A**) and TS mass spectrum (**B**).

**Figure 3:**
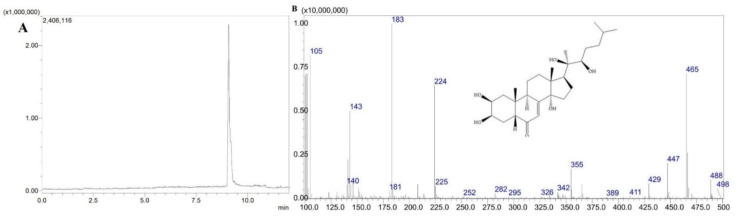
TIC chromatogram (**A**) and mass spectrum of PA (**B**).

Previously established LC–PDA parameters were applied to the LC–MS analysis, with a small modification of the mobile phase involving the inclusion of 0.1% formic acid to increase ionization efficiency. The resulting total ion chromatograms (TICs) demonstrated clear separation and symmetrical peak shapes for 20-HE (retention time (t_R_=6.40 min), TS (t_R_=5.40 min), and PA (t_R_=8.65 min) (Figure 1, Figure 2 and Figure 3). Selected ion monitoring (SIM) provided enhanced sensitivity and selectivity for quantitative analysis.

### 2.1. Validation of the Method

#### 2.1.1. Linearity

Linearity was evaluated using external standard calibration curves constructed from five concentration levels (15–150 ng/mL), each analyzed in triplicate. Excellent linear correlations were obtained for all analytes, with regression coefficients (R^2^) of 0.9998 for TS and calibration equation (y = 267,752x + 2,000,000), 0.9999 for 20-HE with calibration equation (y = 85,584x − 176,652), and 0.9998 for PA with calibration equation (y = 36,439x − 268,761). The high R^2^ values confirm the strong linear relationship between peak area and analyte concentration within the tested range.

#### 2.1.2. Accuracy and Precision

The three-level concentrations used to determine accuracy were as follows. TS: high concentration level—125 ng/mL, medium concentration level—75 ng/mL, and low concentration level—20 ng/mL; 20-HE: high—125 ng/mL, medium—75 ng/mL, and low—20 ng/mL; and PA: high—125 ng/mL, medium—75 ng/mL, and low—20 ng/mL, each measured in four replicates. The percentage accuracy for TS ranges from 100.12 to 100.21%, 99.77 to 100.21% for 20-HE, and 100.06 to 100.21% for PS. The obtained results demonstrate the excellent accuracy of the proposed method. Table 1 presents the results of the accuracy assessment of the tested PEs. Accuracy values ranged from 99.77% to 100.21%, indicating excellent agreement between measured and nominal concentrations. The low coefficients of variation (CV < 0.5%) further demonstrate the reliability of the method. However, such low variability is partly attributable to the use of standard solutions and controlled experimental conditions, which minimize matrix-related effects.

The precision of an analytical method indicates the reproducibility of repeated measurements obtained from the same homogeneous sample under consistent conditions. It was evaluated in terms of intra-day and inter-day precision by calculating the CV values for three known concentration levels, each analyzed in four replicates. Repeatability was estimated as the coefficient of variation for three concentrations with four replicates, and intermediate precision was assessed for three concentrations within three days with four replicates each day. Table 2 presents the precision results of the developed method. Both intra-day and inter-day precision values showed below 1%, confirming the method’s high repeatability and intermediate precision in accordance with International Council for Harmonisation validation guidelines.

#### 2.1.3. Limit of Detection (LOD) and Quantification (LOQ)

The limits were determined based on linearity data by applying the standard deviation of the response together with the slope of the calibration curve. The calculated values were TS—LOD 4.37 ng/mL and LOQ 13.24 ng/mL; 20-HE—LOD 3.78 ng/mL and LOQ 11.45 ng/mL; and PA—LOD 4.64 ng/mL and LOQ 14.05 ng/mL. The low LOD and LOQ values indicate high sensitivity of the method, making it suitable for the determination of PEs in plant matrices where analyte concentrations may vary significantly.

#### 2.1.4. Robustness

In the current study, robustness was assessed by modifying the column temperature. To evaluate the influence of temperature fluctuations, temperatures within the range of 42–48 °C were intentionally applied, and the retention time of each compound was determined. No significant change in resolution was observed with temperature variation. Robustness of the method was further evaluated by introducing small deliberate variations in mobile phase composition (±2% organic modifier) and flow rate (±0.1 mL/min). No significant changes in retention time, peak shape, or resolution were observed under the tested conditions. The obtained results indicate that the method is robust and suitable for reliable quantitative analysis, demonstrating good tolerance to minor variations in analytical parameters and potential applicability across different laboratory settings.

#### 2.1.5. Stability

Standard stock solutions were stored at 2–8 °C. Before analysis, the solutions were visually examined to confirm their clarity and then analyzed using the developed method. A comparison between chromatograms of freshly prepared and stored solutions showed no significant differences, indicating that the samples maintained their stability during the storage period and could be analyzed without chemical degradation. Autosampler stability was evaluated by reanalyzing processed samples after 24 h storage in the autosampler at 15 °C. The measured concentrations were compared with those obtained from freshly prepared samples. The results demonstrated that all three ecdysteroids remained stable under these conditions.

### 2.2. Matrix Effect

To further evaluate the applicability of the method in complex matrices, matrix effects were examined by post-extraction spiking at 75 ng/mL in one representative plant matrix (spinach sample S3) and one representative DS matrix (sample 5). The obtained matrix effect values were 88.49%, 90.74%, and 88.99% for 20-HE, TS, and PA, respectively, in spinach sample S3, and 95.24%, 95.69%, and 96.88%, respectively, in sample 5. These results indicate limited to moderate ion suppression, which was more pronounced in the plant matrix than in the dietary supplement matrix. The observed matrix effects were not negligible, but remained within a range that did not compromise the quantitative applicability of the method under the selected analytical conditions. These findings support the suitability of the developed LC–MS method for routine analysis of PEs in plant extracts and DSs, while also confirming that matrix composition may influence ionization efficiency to some extent.

### 2.3. Application of the Method

**Figure 4:**
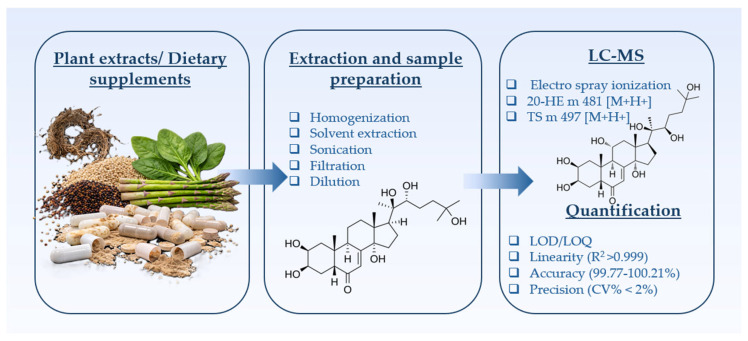
Schematic representation of the analytical workflow for the determination and quantification of PEs in plant extracts and DSs.

To provide a clear overview of the analytical strategy employed in the present study, the overall workflow encompassing plant material and dietary supplement sampling, extraction and preparation procedures, LC–MS analysis, and quantitative validation is summarized in Figure 4. This integrated approach ensures reliable identification and quantification of the principal PEs in complex plant matrices and commercial DSs.

The developed LC–MS method demonstrated excellent linearity, precision, and accuracy. Correlation coefficients were close to unity, and the combination of high accuracy percentages with low standard deviation values confirmed the method’s reliability for the quantitative determination of 20-HE, TS, and PA. Following validation, the method was applied to confirm the results previously obtained using the LC–PDA procedure [8]. Plant extracts from *R. carthamoides* (wild and cultivated), spinach, quinoa, kaniwa, and asparagus were analyzed. Quantitative analysis of ecdysteroids yielded the following results from the LC-MS method, as shown in Table 3.

Analysis of *R. carthamoides* extracts revealed notable differences between wild and cultivated samples. The wild sample contained 2.97 mg/g 20-HE, 1.58 mg/g PA, and 1.72 mg/g TS, whereas the cultivated Bulgarian sample contained 1.72 mg/g 20-HE, 0.40 mg/g PA, and 0.97 mg/g TS. These results indicate substantial variability depending on cultivation conditions and geographical origin. Among the so-called “superfood” samples, the 20-HE contents (expressed per dry weight) were white quinoa—330 µg/g, red quinoa—272 µg/g, kaniwa—691 µg/g, spinach S1—462 µg/g, spinach S2—272 µg/g, spinach S3—260 µg/g, and asparagus—198 µg/g. All measurements were performed in triplicate, and the standard deviation did not exceed 2%; therefore, it is not displayed.

The content of 20-HE in *R. carthamoides* exhibits considerable variability depending on the plant organ, developmental stage, and cultivation conditions. Reported concentrations in roots range from 0.087% to 0.35% of dry mass [35]. A pronounced seasonal dependence has been observed, with values varying between 0.04 and 0.81% in roots, 0.03 and 1.22% in leaves, and 0.27 and 1.51% in seeds [36]. It has been reported that 20-HE content may reach up to 1.42%, with the highest concentrations detected in seeds, in roots at the beginning of the vegetation period, and in aerial parts during the active growth phase [37]. An isolation method for 20-HE from roots and rhizomes has also been described, achieving a yield of approximately 0.05% relative to the raw material [38]. In quinoa seed extract, 20-HE was quantified at 310 µg/g dry weight, consistent with previously reported values of 138–570 µg/g [39,40]. In kaniwa seeds, the content reached 670 µg/g dry weight—substantially higher than the previously reported 15 µg/g [41]. Among the three analyzed spinach samples (S1, S2, and S3), notable variability was observed, with concentrations of 455 µg/g, 266 µg/g, and 252 µg/g dry weight, respectively. These differences reflect variations in cultivation conditions, geographical origin, and developmental stage. The obtained range (252–455 µg/g) aligns with previously published data (79.6–428 µg/g), depending on processing methods [42]. A broader variability (17.1–885 µg/g) has also been reported, and it has been shown that the use of 20% ethanol as an extraction solvent increases 20-HE yield to 91% compared to approximately 80% in aqueous extraction [43,44]. Depending on the cultivar, spinach may contain significantly lower amounts of 20-HE (10.3–50 µg/g dry weight) [3,45,46], with values as low as 10 µg per 100 g of fresh leaves also reported [17]. The observed variability in 20-HE content among the analyzed spinach samples may also be influenced by differences in harvesting time. In the present study, sample S1 was collected during the spring harvest period, corresponding to the active vegetative growth stage of *S*. *oleracea*. It is well established that the accumulation of PEs depends on plant organ, developmental stage, and environmental conditions, which may further contribute to the differences observed between samples. In the asparagus stem extract, the 20-HE content reached 189 µg/g, exceeding some previously published values [3,45]. A low 20-HE concentration (0.52 µg/g) was reported in fresh arugula leaves [42]. The extraction efficiency may vary among different plant matrices, particularly between lipid-rich seeds (e.g., quinoa, kaniwa) and leafy tissues (e.g., spinach). Although the applied extraction protocol yielded consistent and reproducible results across the investigated samples, further optimization of extraction solvents or solvent systems may enhance analyte recovery in specific matrices. Extraction efficiency is known to depend on several parameters, including solvent composition, extraction time, temperature, and number of extraction cycles. In the present study, a consistent extraction protocol was applied to all samples to ensure comparability of the obtained results. Overall, the results demonstrate substantial variability in 20-HE levels among different plant species, organs, and developmental stages. These differences are influenced by biological, environmental, and methodological factors. Therefore, standardization of extraction and analytical procedures is essential to ensure comparability and reliability of results in future studies.

Although 20-HE occurs naturally in foods such as spinach, quinoa, and asparagus [47], any potential regulatory restriction could be implemented through threshold values, analogous to salbutamol and formoterol [48]. Regardless of its future regulatory status, stringent quality control of DSs—particularly when used by athletes—remains imperative. Ecdysteroid-enriched supplements obtained from *R. carthamoides*, *S. oleracea*, *Cyanotis arachnoidea*, and *Pfaffia* species have gained increasing popularity among athletes due to their potential anabolic-like effects [7]. While the dietary intake of ecdysteroids from natural foods seldom exceeds 100 mg per day, supplementation often reaches 100–1000 mg, levels associated with enhanced strength and muscle mass [6,7]. An investigation confirmed the dose-dependent anabolic activity of 20-HE, leading to its inclusion in the 2020 WADA Monitoring Program and consideration for future prohibition [13,49]. DSs are not required to undergo quality testing prior to market release, and manufacturers are not obligated to provide evidence regarding their safety or effectiveness before commercialization [50]. Numerous cases have revealed significant discrepancies between the labeled information of certain DSs and their actual composition. Consequently, there is a need to develop new analytical methods that are rapid, precise, accurate, and sustainable for the evaluation of DSs containing PEs using appropriate techniques [16].

Using the developed LC–MS procedure, thirteen randomly selected dietary supplements labeled as plant extracts of *C. arachnoidea*, *R. carthamoides*, and *A. turkestanica* were analyzed to determine the presence and concentration of 20-HE and TS. The quantified amounts of 20-HE in these samples are listed in Table 4.

Among the investigated products, one sample originating from the Czech Republic and labeled to contain 300 mg of *C. arachnoidea* extract standardized to 90% ecdysterone showed a determined 20-HE content of 107.05 ± 1.07 mg per capsule, while the TS content was 13.78 ± 0.14 mg. The measured ecdysterone amount was substantially lower than the value expected from the declared 90% standardization. Seven products containing *R. carthamoides* extract were evaluated. Considerable variability between labeled and determined content was observed. A product labeled to contain 250 mg extract without specified standardization showed 105.03 ± 0.32 mg 20-HE and 0.32 ± 0.03 mg TS. In contrast, a product labeled as containing 95% ecdysterone (245 mg extract) contained only 21.80 ± 0.21 mg 20-HE and 0.80 ± 0.08 mg TS. Similarly, a preparation labeled as 80% ecdysterone (250 mg extract) showed 80.09 ± 0.80 mg ecdysterone and 19.80 ± 0.19 mg TS, values not corresponding to the declared percentage. Lower-dose tablet formulations (35 mg and 40 mg extract) demonstrated 20-HE contents of 14.50 ± 0.15 mg and 16.02 ± 0.09 mg, respectively, which were relatively consistent with the labeled amounts (15 mg and 16.5 mg). However, TS levels in these products were minimal (≤0.17 mg). One product containing 200 mg extract showed only 4.19 ± 0.05 mg 20-HE, with TS below the LOD. Another capsule product (60 mg extract) demonstrated both 20-HE and TS below LOD. A high-dose product labeled to contain 600 mg extract standardized to 95% 20-HE contained 49.98 ± 0.05 mg 20-HE, markedly lower than expected, while TS was not detected. Four products containing *A. turkestanica* extract standardized to 10% TS were analyzed. One sample showed 54.77 ± 0.54 mg TS with minimal 20-HE content (0.47 ± 0.04 mg), corresponding to TS enrichment. However, other products displayed discrepancies between labeled and measured values. One preparation contained 24.98 ± 0.34 mg 20-HE and only 0.64 ± 0.06 mg TS, whereas another showed 2.5 ± 0.04 mg 20-HE and 6.23 ± 0.08 mg TS. The highest combined 20-HE and TS levels among these products were observed in a sample containing 35.51 ± 0.35 mg 20-HE and 40.03 ± 0.40 mg TS. In several cases, the discrepancies between labeled and measured content were particularly pronounced. For example, a product labeled as containing 95% ecdysterone showed only 21.80 ± 0.21 mg of 20-HE per capsule, which is markedly lower than expected based on the declared standardization. Similarly, another product labeled as 95% ecdysterone contained only 49.98 ± 0.05 mg per capsule. These findings highlight substantial inconsistencies between declared and actual content. Significant inconsistencies were identified between the declared and experimentally determined content of ecdysteroids in the analyzed DSs. Some products labeled as highly standardized extracts contained substantially lower amounts of active compounds than stated, whereas certain non-standardized products exhibited moderate to high levels of ecdysterone. Similar findings have been reported by Ambrosio et al. and Kraiem et al., who likewise observed inconsistencies between labeled and actual content [25,51]. Furthermore, it has been reported that supplements marketed as containing high concentrations of TS in fact contained higher amounts of 20-HE [25]. These discrepancies may reflect limitations in quality control and labeling accuracy. However, other factors should also be considered. In particular, the chemical stability of TS and 20-HE during processing and storage may influence their measured concentration, as degradation under conditions such as exposure to heat, light, oxygen, or prolonged storage cannot be excluded. Nevertheless, the observed variability highlights the absence of mandatory analytical control of DSs within the European Union and raises concerns regarding labeling reliability and extract quality. The implementation of stricter regulatory mechanisms and adherence to Good Laboratory Practice standards appear warranted. Given the rapid expansion of the dietary supplement market over the past two decades [52], ensuring effective quality control has become increasingly important.

Interest in 20-HE is driven by published data suggesting potential anabolic effects. At present, TS is not included in the WADA Monitoring Program or the Prohibited List. Nevertheless, in light of its potential biological effects and increasing use, future regulatory changes cannot be excluded. The development and validation of sensitive and rapid analytical methods are essential both for quality control of DSs and for anti-doping purposes [24]. Moreover, in the European Union, DSs are regulated as foods under Directive 2002/46/EC and Regulation (EC) No. 1924/2006 [53,54]. However, pre-market verification of active compound content is not mandatory, which may contribute to the discrepancies observed between labeled and experimentally determined ecdysteroid concentrations [53,54]. In addition, certain less common plant-derived ingredients used in supplements, such as kaniwa, may be considered novel foods under Regulation (EU) 2015/2283, subject to case-by-case assessment based on their history of consumption in the European Union prior to 15 May 1997 [55]. This regulatory context may further contribute to variability in product composition and standardization across commercially available supplements.

The LC–MS method developed in the current study provides a rapid and cost-effective alternative using a single–quadrupole mass spectrometer while maintaining excellent selectivity, sensitivity, and reproducibility. Unlike previous approaches, the method enables the simultaneous quantification of three major PEs (20-HE, TS, and PA) across both nutrient-dense plant matrices and commercial DSs within a single analytical workflow. The method demonstrated robust performance across diverse matrices, confirming its suitability for quantitative analysis. Its simplicity and analytical efficiency make it particularly suitable for routine screening and quality control of ecdysteroid-containing products. The method represents a rapid analytical platform suitable for routine control of ecdysteroids across diverse matrices. Future studies should consider the use of isotopically labeled internal standards to compensate for potential ion suppression or enhancement effects in complex matrices.

## 3. Materials and Methods

### 3.1. Plant Materials and Extracts Preparation

Fresh spinach leaves (*Spinacia oleracea* L., Chenopodiaceae) used for samples S2 and S3, quinoa seeds (*Chenopodium quinoa* Willd., Chenopodiaceae—white and red varieties), kaniwa seeds (*Chenopodium pallidicaule*, Chenopodiaceae), and asparagus stems (*Asparagus officinalis* L., Asparagaceae) were obtained from local organic markets. Spinach leaves used for sample S1 were collected in the village of Belashtitsa, located in the Thracian Lowland floristic region of Bulgaria (42.064729, 24.751886) during the spring harvest period (May). Roots and rhizomes of wild-growing *R. carthamoides* were sourced from Siberia, Russia, whereas cultivated specimens of *R. carthamoides* were collected from plants grown in Bulgaria. The extracts were prepared following the previously reported procedure [9]. Plant samples were ground using a mortar and pestle and extracted with methanol–water (50:50, *v*/*v*) at a solvent-to-sample ratio of 20:1, *v*/*w* and subjected to ultrasonication for 30 min in an ultrasonic bath (Bandelin, Berlin, Germany) at room temperature. The resulting extracts were filtered through a 0.45 µm membrane filter and stored in amber vials at 4 °C until analysis. All samples were prepared and analyzed in triplicate.

### 3.2. Chemicals and Reagents

Reference standards of 20-hydroxyecdysone (molecular weight 480.64 g/mol, purity: HPLC ≥ 95%, #89,651) and turkesterone (molecular weight: 496.6 g/mol, purity: HPLC ≥ 95%, #85,781) were acquired from PhytoLab GmbH & Co. KG (Vestenbergsgreuth, Germany). Ponasterone A (molecular weight: 464.6 g/mol, purity: HPLC ≥ 95%, #16,386) was sourced from Cayman Chemical (Ann Arbor, MI, USA). Acetonitrile suitable for LC–MS analysis was provided by Merck KGaA (Darmstadt, Germany).

### 3.3. Dietary Supplements

Thirteen randomly selected dietary supplement products were obtained through online purchasing and included in the study. Tablet and capsule samples were first ground to a homogeneous powder and accurately weighed prior to dissolution. For each product, three individual capsules or tablets were analyzed independently. Extraction was performed using 10 mL of acetonitrile, followed by ultrasonication for 15 min. Acetonitrile was selected as the extraction solvent due to its compatibility with LC–MS analysis and its ability to efficiently extract PEs. Ultrasonication was applied to enhance extraction efficiency. The resulting solutions were filtered through a 0.45 μm syringe filter and subsequently diluted to an appropriate volume before injection into the analytical system. All samples were prepared in triplicate. Dietary supplement samples were prepared by grinding tablets and capsule contents to a homogeneous powder, followed by extraction with 10 mL acetonitrile and ultrasonication for 15 min. The resulting solutions were filtered through a 0.45 μm syringe filter and diluted to an appropriate volume before injection. All samples were prepared and analyzed in triplicate.

### 3.4. Preparation of Standard Solutions

Stock solutions of the reference compounds 20-HE, PA, and TS were prepared at a concentration of 1 mg/mL using an acetonitrile–water mixture (50:50, *v*/*v*) to obtain five calibration levels in the concentration range of 15–150 ng/mL, following a previously described procedure [8]. To ensure complete dissolution, the solutions were sonicated in an ultrasonic bath (Bandelin, Berlin, Germany). The prepared stock solutions were kept in light-protected vials and stored at 4 °C until analysis. Working standard solutions were subsequently obtained by diluting the stock solutions with the same acetonitrile–water (50:50, *v*/*v*) mixture to produce a range of concentrations.

### 3.5. Instrumentation

The analytical method was established using a Shimadzu LCMS-2050 liquid chromatography–mass spectrometry system (Shimadzu, Kyoto, Japan). Separation was carried out on a Shim-pack C18 column with dimensions of 150 mm × 4.6 mm and a particle size of 3 μm.

### 3.6. Chromatographic and Mass Spectrometer Conditions

The used chromatographic conditions (the column, operating temperature conditions, and gradient elution) are according to the previously reported protocol by Todorova et al. [8], with slight modification of the mobile phase by the addition of 0.1% formic acid to improve ionization efficiency. The mobile phase consisted of solvent A, water containing 0.1% formic acid, and solvent B, acetonitrile. Gradient elution was performed as follows: initial conditions 80% A and 20% B at a flow rate of 0.4 mL/min; at 4 min, the flow rate was increased to 0.5 mL/min and the proportion of solvent B was increased to 50%; at 7 min, the proportion of solvent B was adjusted to 40%; and at 10 min, the system was returned to the initial conditions. The total run time was 12 min. The column temperature was maintained at 45 °C throughout the analysis. The injection volume was 10 μL. Data acquisition and processing were carried out using LabSolutions software (version 5.118) (Shimadzu, Kyoto, Japan). For the analysis, the MS detector was equipped with a single quadrupole. The flow rates of the nebulizing, drying, and heating gases were 2 L/min, 5 L/min, and 7 L/min, respectively. The desolvation temperature was 450 °C. The DUIS voltage interface 3 kV was utilized. The mass spectrometer was operated in electrospray ionization—positive mode. The mass range is from 100 to 500 m/z for TIC. The SIM was applied with the following ions: for 20-HE, 481, 445, 427, and 347; for TS, 497, 479, 462, and 443; and for PA, 465, 429, 224, and 183. The retention times were approximately 6.40 min for 20-HE, 5.40 min for TS, and 8.65 min for PA. Data acquisition and processing were performed using LabSolutions software. All samples and standard solutions were analyzed in triplicate under the same instrumental conditions to ensure reproducibility of the analytical procedure.

### 3.7. Validation of the Method

Method validation was carried out in accordance with the International Council for Harmonisation (ICH) recommendations for analytical method validation, assessing the following parameters: linearity, range, precision, accuracy, LOD, LOQ, and robustness [56].

### 3.8. Matrix Effect Assessment

Matrix effects were assessed using a post-extraction spiking approach at a representative concentration level of 75 ng/mL. Two representative matrices were selected: spinach sample S3, as the plant sample with the lowest endogenous 20-HE content, and DS—sample 5. Extracts were prepared according to the procedure described above. Aliquots of the final extracts were spiked after extraction with mixed standard solutions of 20-HE, TS, and PA. All measurements were performed in triplicate under the same instrumental conditions. Matrix effect (%) was calculated by comparing the response of post-extraction spiked samples with that of standard solutions.

## 4. Conclusions

A reliable and efficient LC–MS method was developed and validated for the simultaneous quantification of 20-HE, TS, and PA in plant-derived extracts and DSs. The method demonstrated strong linearity, good precision and accuracy, and low detection limits, confirming its suitability for quantitative analysis of PEs across diverse matrices. Application of the method confirmed nutritionally important foods such as quinoa, kaniwa, spinach, and asparagus as natural sources of PEs, with pronounced variability in 20-HE levels among species and samples. Significant differences were also observed between wild and cultivated *R. carthamoides*, indicating that geographical origin and cultivation conditions strongly affect PE accumulation. Analysis of thirteen commercially available DSs revealed substantial discrepancies between labeled and experimentally determined ecdysteroid content. Several products marketed as highly standardized contained markedly lower amounts than declared, while some non-standardized products showed moderate to high levels. Considerable variability in TS content was likewise identified among products labeled as standardized *A. turkestanica* extracts. These findings highlight the need for improved quality control and more reliable labeling of ecdysteroid-containing products, particularly in the context of their increasing use in sports nutrition. While the method demonstrates strong analytical performance for plant extracts and oral dosage forms, further validation in biological matrices, such as urine and plasma, would be required before its application in anti-doping monitoring.

## Figures and Tables

**Table 1:** Accuracy of the developed LC-MS method.

Concentration (ng/mL)	Average Concentration (ng/mL ± SD)	Accuracy%	CV%
**Turkesterone**			
125	125.17 ± 0.21	100.13	0.17
75	75.16 ± 0.21	100.21	0.28
20	20.02 ± 0.08	100.12	0.40
**20-Hydroxyecdysone**			
125	125.26 ± 0.45	100.21	0.36
75	74.98 ± 0.13	99.98	0.18
20	19.95 ± 0.07	99.77	0.38
**Ponasterone A**			
125	125.27 ± 0.35	100.21	0.28
75	75.04 ± 0.22	100.06	0.29
20	20.02 ± 0.09	100.11	0.43

**Table 2:** Precision of the developed method.

Concentration (ng/mL)	Intra-Day Precision			Inter-Day Precision		
	Mean (ng/mL ± SD)	SEM	CV%	Mean (ng/mL ± SD)	SEM	CV%
**Turkesterone**						
125	124.94 ± 0.40	0.16	0.32	125.20 ± 0.57	0.23	0.46
75	75.15 ± 0.25	0.10	0.34	75.14 ± 0.58	0.24	0.77
20	19.97 ± 0.09	0.03	0.43	20.00 ± 0.11	0.04	0.54
**20-Hydroxyecdysone**						
125	125.26 ± 0.45	0.18	0.36	125.28 ± 0.57	0.23	0.45
75	75.07 ± 0.15	0.06	0.21	75.11 ± 0.24	0.09	0.32
20	19.96 ± 0.08	0.03	0.39	19.93 ± 0.15	0.06	0.73
**Ponasterone A**						
125	126.03 ± 0.81	0.33	0.64	125.84 ± 0.88	0.36	0.69
75	74.86 ± 0.26	0.10	0.35	74.86 ± 0.32	0.13	0.42
20	20.03 ± 0.09	0.03	0.43	20.05 ± 0.12	0.05	0.57

**Table 3:** Content of PEs in analyzed plant samples.

Sample	Plant Material	20-HE	TS	PA
*R. carthamoides* (wild population)	Roots and rhizomes	2.97 mg/g	1.72 mg/g	1.58 mg/g
*R. carthamoides* (cultivated population)	Roots and rhizomes	1.72 mg/g	0.97 mg/g	0.40 mg/g
White quinoa	Seeds	330 µg/g	–	–
Red quinoa	Seeds	272 µg/g	–	–
Kaniwa	Seeds	691 µg/g	–	–
Spinach S1	Leaves	462 µg/g	–	–
Spinach S2	Leaves	272 µg/g	–	–
Spinach S3	Leaves	260 µg/g	–	–
Asparagus	Stems	198 µg/g	–	–

All values are expressed as means of triplicate analyses; RSD < 2%.

**Table 4:** Measured concentrations of 20-HE and TS in analyzed DSs.

Sample No.	Country of Origin of the Manufacturer	Product Description	Labeled Extract Content (mg/Capsule or Tablet)	20-HE/TS Labeled Content	Measured 20-HE Content (mg ± SD/Capsule or Tablet)	Measured TS Content (mg ± SD/Capsule or Tablet)
1	Bulgaria	β-ecdysterone from *R. carthamoides* extract	245 mg per capsule	95% ecdysterone	21.80 ± 0.21 mg	0.80 ± 0.08 mg
2	Bulgaria	*R. carthamoides* extract	200 mg per tablet	Not labeled	4.19 ± 0.05 mg	<LOD
3	Bulgaria	*R. carthamoides* extract	35 mg per tablet	15 mg	14.50 ± 0.15 mg	0.17 ± 0.07 mg
4	Bulgaria	*R. carthamoides* extract	40 mg per tablet	16.5 mg	16.02 ± 0.09 mg	0.09 ± 0.02 mg
5	Bulgaria	*R. carthamoides* extract	60 mg per capsule	Not labeled	<LOD	<LOD
6	Bulgaria	*R. carthamoides* extract	600 mg per capsule	95% ecdysterone	49.98 ± 0.05 mg	<LOD
7	Czech Republic	*C. arachnoidea* extract	300 mg per capsule	90% ecdysterone	107.05 ± 1.07 mg	13.78 ± 0.14 mg
8	Not mention	*R. carthamoides* extract	250 mg per capsule	Not labeled	105.03 ± 0.32 mg	0.32 ± 0.03 mg
9	Not specified	*R. carthamoides* extract	250 mg per capsule	80% ecdysterone	80.09 ± 0.80 mg	19.80 ± 0.19 mg
10	Sweden	*A. turkestanica* extract + ginseng and astragal extract	500 mg per capsule	Min. 10% turkesterone	0.47 ± 0.04 mg	54.77 ± 0.54 mg
11	Bulgaria	*A. turkestanica* extract	500 mg per capsule	10% turkesterone	24.98 ± 0.34 mg	0.64 ± 0.06 mg
12	American brand, with the country of production not specified on the label	*A. turkestanica* extract	500 mg per capsule	10% turkesterone	2.5 ± 0.04 mg	6.23 ± 0.08 mg
13	Bulgaria	*A. turkestanica* extract	600 mg per capsule	10% turkesterone	35.51 ± 0.35 mg	40.03 ± 0.40 mg

The results are presented in triplicate, and the RSD does not exceed 2%.

## Data Availability

The original contributions presented in this study are included in the article. Further inquiries can be directed to the corresponding author.

## Abbreviations

The following abbreviations are used in this manuscript:

20-HE	20-hydroxyecdysone
CV	Coefficient of variation
GC–MS	Gas chromatography–mass spectrometry
HPLC	High-performance liquid chromatography
LC-MS	Liquid chromatography–mass spectrometry
LOD	Limit of detection
LOQ	Limit of quantification
PA	Ponasterone A
PEs	Phytoecdysteroids
SIM	Selective ion monitoring
TIC	Total ion chromatogram
TS	Turkesterone
